# Selections and Abstracts

**Published:** 1905-08

**Authors:** 


### SELECTIONS AND ABSTRACTS.

### Talk and Demonstration of the Manner of Preparation of Various Articles Intended for Nourishment of the Sick.

By Miss C. Wooster, Fort Wayne.

Of all the advancements during the nineteenth century few have had a greater influence for good than the progress in scientific cooking.

Disease is often due to improper feeding. Rations often do not contain the correct proportions of food principles or the food is improperly cooked. In order to prescribe the needed rations to his patient a doctor should make a careful study of the digestibility and particular nutritive value of the various foods, and the best methods of preparing these foods. Some proper foods may be made very unwholesome by improper cooking. Foods are cooked to develop flavor, increase palatability, to assist digestion and to destroy germs.

Nature has provided two perfect foods which contain the necessary nutriment in proper proportions; these foods are milk and eggs. Milk, however, is a perfect food only for an infant, and is not most suitable for an adult. In comparing the composition of cow's milk and human milk, it will be seen that cow's milk contains more proteid and mineral matter and less sugar of milk than does human milk. The proteid of milk is composed of lactalbumin and caseinogen; the former, soluble in water, makes a large per cent. of the albumen of human milk. Human milk forms into floculent curds during digestion, whereas cow's milk, containing more caseinogen, forms into larger, denser curds.

When an infant must be given cow's milk, it should be modified. However, when the milk is procured from a reliable source, sterilization and pasteurization are necessary only in cases of bowel trouble and in hot weather.

Home modification has advantages over laboratory modification.

Procure the milk as fresh as possible, reduce its temperature to 45 degrees Fahrenheit and allow to stand six to eight hours for the cream to rise, and pour off the desired amount of top milk. It may now be modified as follows:

| | |
|---|---|
| Top milk (10 per cent fat) | 8 ounces |
| Boiled water | 11 ounces |
| Lime water | 1 ounce |
| Milk sugar | 2½ tablespoonfuls |

Dissolve the sugar in the water and pour in the milk and lime water. Put the mixture into ten sterile nursing bottles, plug them with cotton and set them in a cool place till needed. The modified milk may be sterilized by steaming it in a steamer 45 minutes. If it is to be thus sterilized it is not necessary to use boiled water. Before using the milk it should be warmed lukewarm by placing in warm water.

Regular feeding should be insisted upon. The following table is an excellent one for infant feeding:

From birth to 2 months old, 10 feedings per day, at 3, 6, 8, 10 and 12 a. m. and 2, 4, 6, 8 and 10 p. m.

Two to 3 months, 8 feedings per day, at 6, 9 and 12 a. m. and 2, 4, 6, 8 and 10 p. m.

Three to 6 months, 6 feedings per day, at 6, 9 and 12 a. m. and 3, 6 and 10 p. m.

Six to 12 months, 5 feedings per day, at 6, 9 and 12 a. m. and 3 and 6 p. m.

Twelve to 16 months, 4 feedings per day, at 7:30 and 11:30 a. m. and 2:30 and 7:30 p. m.

Feeding in health is of great importance, and in diseases it becomes a question of supreme moment. The teeth, mouth, stomach and entire alimentary canal must be considered, for the food to be of value must be assimilated. An invalid's tray should be set with special care as to neatness, cheer and cleanliness. Take care to have cold foods served cold and hot foods served hot. Serve the water in small quantities and let it be freshly drawn.

Beef Tea—Beef tea contains the juices of beef diluted with water. It is given as a stimulant and not as a nutrient, as is generally supposed. The stimulating property of beef tea is due to creatin, which closely resembles the thein in tea and the caffein in coffee, both in composition and in its effect upon the nervous system. Meat extractives are the greatest known stimulants to the secretions of gastric juice.

Many preparations made from beef are on the market. They are not as numerous nor yet as palatable as carefully prepared home-made beef tea. Beef for the making of beef extract and beef tea should be cut from the round of a heavy corn-fed steer to insure good flavor and a large quantity of juice. Remove the fat and cut into small pieces one-half pound of this meat; put it into a sterile glass jar, cover the jar and place it on a trivet in a kettle of cold water; heat the water gradually and keep it at 130 degrees Fahrenheit for two hours. Turn the meat from the jar and press out the juices. Season with salt and reheat the juice in a cup set in a pan of watar. Do not heat it hot enough to coagulate the albumen.

Beef Tea No. 1—Dilute beef extract and an equal quantity of hot water.

Beef Tea No. 2.—One pound of beefsteak and two cups cold water. Prepare as follows: Put the finely cut beef and the water into a glass jar for twenty minutes. Now heat on trivet in kettle of water at 130 degrees for two hours, then slightly increase the temperature to coagulate the albumen. This extra heating will destroy the raw taste otherwise present.

Rice and Potatoes—Rice is exceedingly rich in starch and contains a small amount of proteid, fat and mineral matter. It contains four times as much nourishment as potatoes and requires only one hour for its complete digestion. It is readily absorbed and leaves little or no waste in the intestines. Rice is a carbohydrate, a fat-forming and an energy-producing food. The digestion of rice begins in the mouth and is completed in the intestines.

How to Steam Rice—Take three tablespoonfuls of rice, one cup boiling water and one-quarter teaspoonful of salt. Add the rice to the boiling water, boil three minutes, then place the containing vessel in a larger one of boiling water and thus steam for one hour.

Add salt and serve with sugar and cream. The rice may be cooked with half water and half milk.

Potatoes are a rather inexpensive and a very popular food. They appear on our table from once to three times daily. They contain very small quantity of celulose and if properly cooked are easily digested. The preferred method of cooking potatoes for a young child or for an invalid is to bake them in a hot oven. This changes some of the starch to dextrin and increases their digestibility. If baked in a slow oven they have no advantage over boiled potatoes. Too hot an oven hardens the skin, prevents evaporation and makes them dark and soggy. To bake potatoes, place them in a hot oven until they are soft, press on them to rupture the skin and let out the water as steam. Now serve at once before they reabsorb moisture. Serve with butter or cream and salt.

Function of Proteids—The proteids are divided into albumins, gelatins and extracts. The principal animal foods containing proteid are meat, eggs and cheese. Vegetables containing much proteid are cereals, beans, peas and lentils. Albumins build up and repair the tissues. Upon oxidation they furnish heat and energy, and under certain conditions they are fat-producing. The gelatins have practically the same uses as the albumins, but in less degree. They tend to build up the circulating rather than the tissue albumin, and in this way prevent tissue consumption.

Extractives act upon the nervous system. While they are not foods themselves, they are essential as regulators of digestion and assimilation.

Eggs furnish a valuable concentrated food, but are deficient in carbohydrates. Therefore, they are well adapted for use with starchy foods, which furnish the necessary bulk. Eggs are principally digested in the stomach. A raw egg is so bland that it does not properly stimulate the flow of gastric juice, consequently it does not leave the stomach so soon as does a more stimulating soft-boiled egg. Two soft-cooked eggs leaves the stomach in one and three-fourths hours. Two raw eggs leave the stomach in two and one-fourth hours. A hard-boiled egg is powdery and quickly acted upon by the gastric juice.

To Cook Eggs Soft—Place the eggs in a vessel of boiling water and remove the water to the back of the stove, where it will not boil. In six to eight minutes take out the eggs, break them into a warm cup, salt and serve hot.

Beef—Raw meat is more digestible, but less palatable than cooked meat. Beef should be bright red in color, fine-grained and well mottled with fat. The digestibility of all meats depends upon the length and thickness of the individual fibers, the quantity of fat between the fibers and the hardness and denseness of the connective tissues.

To Make Beef Balls—Cut the steak into half-inch strips and scrape each separately to remove the soft part of the meat. Form the scrapings into balls. Cook till the balls are seared and serve on buttered toast.

Beverages—Tea, coffee and cocoa are frequently used. These each contain an alkaloid, to which it owes its stimulating property. These alkaloids are thein in tea, caffein in coffee and theobromin in cocoa.

In black tea the leaves are allowed to ferment; in green tea the leaves are unfermented. Tea should always be made as an infusion. Do not allow the tea to boil nor the leaves to be reused. The thein is so soluble that it is at once dissolved out of the leaf. Tannic acid is developed upon boiling the tea. Therefore, steep the tea only for a few moments.

To Make a Pot of Tea—Take three teaspoonfuls of tea, two cups of hot water. Let it stand three to five minutes; strain and serve at once.—*Medical and Surgical Monitor.*

---

### SOME NEGLECTED SYMPTOMS OF NON-SURGICAL GYNECOLOGY.

BY JOHN A. HALE, M.D.,
Alto Pass, Ill.

It is but a lack of inquisitiveness on the part of the general practitioner that has brought about a condition of things in gynecological practice that warrants the assertion so often reiterated in current surgical literature that "Modern gynecology belongs, practically, to the field of operative surgery."

The successful physician, with a characteristic personality of inquisitiveness, can boldly refute such assertions and substantiate his reputation by the thankfulness of a happy clientele of womankind released from a thraldom of suffering by his inquisitiveness.

Diseases of the female organs of generation are more common than any but a physician can suppose, and surgical gynecology has become a necessity from an early neglect of backaches, spineaches and headaches, followed by irregular, scanty, painful, delayed or suppressed menstruation during girlhood. The inquisitive physician rushes not into instrumental interference, nor sends such patients to certain specialists for officious mutilation, but first a volley of seek-farther questions at the patient which elicit the information that such patient passed her days of approaching puberty in an overcrowded public school, or worse, in a jail-like boarding-school for young ladies, adding fuel to the fire of antagonism between brain and indigestible foods, the body growth lags behind, leaving the imprint of the unequal struggle on the reproductive organs.

With poorly established sexual functions and a perfect disregard for menstrual week, the undeveloped woman leaves school to plunge into a vortex of social dissipation, followed later by an assumption of wifely duties and responsibilities toward a husband who has seen only her bewitching face and not her frail body.

Is it hard to fathom the reason why so many such wives at first tolerate marriage obligations and later resent and loath them when the poor, broken-down sexual system refuses longer to continue functions for which it was made, but carelessly unfitted?

Is not such a condition a cause for dread of maternity on the part of the woman which often leads to criminal abortion, with all its attendant sequences?

To the inquisitiveness of the successful physician must be added a power of positiveness, wherein he may teach both the husband and the wife something they should know before their carelessness brings about these later conditions which require the necessity of mutilation.

The woman suffering from continued nervousness, weariness, wakefulness, headache and backache needs the services of a physician, and not a surgeon. Likewise such symptoms as scanty, painful, delayed and suppressed menstruation should be under the care of a physician and not an over-zealous surgeon. Prolapsus, leucorrhea, ulcerations, chronic inflammations, congestions and enlargements are purely the outcome of neglect of just such symptoms as named. The first-named symptoms are but the assertions of Nature that she is tired of the unequal load, and if not relieved she will resist no longer, come what will.

A judicious investigation of seemingly insignificant details and close application to the technique of examination in the early stages of such cases will reveal constipation, congested mucus lining of the vagina, and irritable bladder, with diffuse hyperemia of all pelvic structures and loss of organic or respiratory rythm; that subtle thrill which extends over the whole body synchronous with the beating of the heart and motion of the lungs, plainly perceptible to the trained eye looking upon healthy pelvic viscera. Quick must be the relief of this engorgement, with its pernicious nutrition of the parts and concomitant accumulation of excrementitious matter.

First and foremost in the treatment of this condition comes the remedy of absolute rest to the parts, and then, but no less important, is the removal of improper dress and the re-establishment of abdominal breathing to restore proper circulation of the pelvic viscera. Treatment for the removal of constipation is self-suggestive; rest we can enjoin upon our patient, and abdominal breathing we may advise, but all animal cells, whether single or united in tissues or in organs, consume a certain amount of matter, and those chemical changes by which material brought to the tissues and organs by the blood and transformed into other product through the activity of the living cells which liberation of life energy must be maintained by a continued inherent thrill or respiratory rythm and constant supply of chemical products. This same chemical agent must not induct a destructive blood metamorphosis, but supply food for the debilitated vitality. For such action we must seek some combination of the old and well-tried remedies of ergot and apium, with acceptable hemagogues.

The questionable action heretofore exhibited by various preparations of such remedies has been due, as clinically proven, to the component resinous compounds of the apium in the combination. In Ergoapiol (Smith) the active principles of apium have been isolated and with ergot made to form an acceptable and agreeable compound with invigorating hemagogues, proving of unquestionable benefit in such conditions as mentioned in this article.

When the general practitioner awakens to his responsibility, we will have less of these conditions, a continuation of which invariably produces a capillary varicosis, with its train of evils, manifested more frequently by copious and disagreeable discharges called leucorrhea. But even as late as in this last-named condition the physician will learn that Ergoapiol (Smith) judiciously, consistently and determinedly administered, will prevent much needless mutilation by effecting a cure.

Pre-emption of space for case reports on this subject would scarcely be justifiable, when each reader may cluster the facts as herein stated around well-known principles and evolve therefrom a rational solution of treatment for diseases involving the female genitalia.—*Medical Herald, of St. Joseph, Mo.*

---

### THE INTERNATIONAL MEDICAL CONGRESS.

The next International Medical Congress will be held in Lisbon, April 19 to 26, 1906. It is expected that it will be one of unusual importance, for a meeting which will be held in what has always been considered as an out-of-the-way country. Already the titles of papers from some of the most distinguished men of the medical profession have been received. Some of the topics for discussion that have been selected by the Executive Committee are the following:

Section of Descriptive and Comparative Anatomy, Anthropology, Embryology and Histology.

Definition, structure and composition of protoplasm.

Origin, nature and classification of pigments.

Cellular changes in normal tissues.

Evolution and involution of the thymus gland.

Section of Physiology.

The role of leucocytes in nutrition.

The thyroid secretion.

Renal permeability.

The nutritive value of alcohol.

The physiology of the cytotoxins.

The blood ferments.

Section of General Pathology, Bacteriology and Pathological Anatomy.

What are the present scientific proofs of the parasitic nature of neoplasms, especially of cancer?

Preventive inoculations against bacterial diseases.

Preventive inoculations against protozoic diseases.

Preventive inoculations against diseases from an unknown specific agent.

The pancreas and fat necrosis.

Therapeutics and Pharmacology.

Local therapeutics in infectious diseases.

Separation, from a physiological and therapeutic point of view, of the different radiations produced in Crooke's tubes, those which are sent out by radioactive bodies.

The therapeutic value of bactericidal serums.

The relation between the molecular constitution of organic bodies, and their physiological and therapeutic action.

Section of Medicine.

The pathogenesis of diabetes.

The pathogenesis of arterial hypertension.

The treatment of cirrhosis of the liver.

Cerebrospinal meningitis.

International defense against tuberculosis.

Meningeal hemorrhage.

Section of Pediatrics.

Spastic affections of infancy; classification and pathogenesis.

Cerebrospinal meningitis; etiology and treatment.

The social struggle against rickets.

Orthopedic surgery in affections of nervous origin, spastic and paralytic.

Congenital dislocation of the hip.
The treatment of abdominal tuberculosis (peritoneal.)

Neurology, Psychiatry and Criminal Anthropology.
Penal reform from the anthropologic and psychiatric point of view.
Forms and pathogenesis of dementia praecox.
The relations of progressive muscular atrophy to Charcot's disease.
Cerebal localization in mental disease.
Education and crime.
Stigmata of degeneration and crime.

Section of Surgery.
Septic peritoneal infections; classification and treatment.
Gastrointestinal and intestino-intestinal anastomoses.
Recent additions to arterial and venous surgery.

Section of Medicine and Surgery of the Urinary Organs.
Surgical intervention in Bright's disease.
Surgical treatment of prostato-vesical tuberculosis.
Progress of urology in the diagnosis of renal disease.
Painful cystides.

Section of Ophthalmology.
Blepharoplasty.
Serotherapy in ophthalmology.

Section of Laryngology, Rhinology, Otology and Stomatology.
Study of the epileptogenous action of foreign bodies in the ear, and of vegetations in the naso-pharynx.
The different forms of suppuration of the maxillary sinus.
Injections of paraffin in rhinology.
Differential diagnosis of tubercular, syphilitic and cancerous lesions of the larynx.
Choice of anesthesia in the extraction of teeth.
Treatment of alveolar suppuration.

Section of Obstetrics and Gynecology.
Conservative surgery of the ovaries.
Taberculosis of the adnexa.
Symphisiotomy.

Pregnancy and cancer of the uterus.
Therapy of puerperal infections.

Section of Hygiene and Epidemiology.
The intermediary of yellow fever.
The cooperation of nations to prevent the importation of yellow fever and the pest.
Watering the streets as a means against tuberculosis.
Recent additions to the etiology and epidemiology of epidemic cerebrospinal meningitis.

Section of Military Medicine.
Portable ration of the soldier during campaign.
The purifying of the country water.
Emergency hospitals on the battlefield.

Section of Legal Medicine.
Signs of death from drowning.
Ecchymoses in legal medicine.
Epilepsy in legal medicine.
Organization of medico-legal services.

Section of Colonial and Naval Medicine.
Etiology and prophylaxis of beri-beri.
Etiology and prophylaxis of dysentery in hot countries.
Mental diseases in tropical countries.
Hospital ships and their functions in time of war.
Tuberculosis in the navy and its prophylaxis.

---

### PROGNOSIS AND TREATMENT OF CHRONIC NEPHRITIS.

BY A. C. MORGAN, M.D., OF PHILADELPHIA.

From our knowledge of the pathology of chronic nephritis we can see that the damage done to the kidney can not be repaired, says Dr. Morgan, therefore we must direct our treatment toward preventing or inhibiting advance of the pathologic process, and upon the results of this treatment the prognosis will largely depend.

Only a small proportion of patients suffering from chronic interstitial nephritis die from this cause alone, complications supervening to terminate the case in a majority of instances.

Chief among these are the pneumonias, especially bronchopneumonia, la grippe, cerebral hemorrhage, cardiac dilatation and inflammation of serous membranes, pericarditis being perhaps the gravest of the latter conditions.

Uremia, specially evidenced by convulsions, is a positive indication that failure of compensation of the kidney has occurred and the prognosis here is grave. Compensation may be restored one or more times, but the patient is badly crippled, and never regains his former degree of health, but goes downward, finally, after a year or so, suffering his final attack of uremia or other complication, or dying of asthenia.

In the treatment of chronic nephritis much can be done by treating the patient and not the disease, endeavoring always at once to remove the cause, wherever known.

As chronic intersital nephritis exists for years before its symptoms manifest themselves, or is accidentally discovered, proving that there is a long period of perfect compensation, diuretics should never be used until there is actual need for them. The same general rules as obtain in compensated heart murmurs should apply with equal force in this disease. Once the diagnosis is made the cardinal rule of "moderation" should govern us in considering the case in hand.

Some general rules as to hygiene are essential. The patient should wear flannels the year round, that the skin action may be active and steady, and to protect him from marked changes of weather. When possible, the winter should be passed in a warm, equable climate.

A country life, rather than seashore, is probably better for the patient, as in the former environment there is less temptation and opportunity to indulge in the many dissipations of seashore life, such as social events, irregular hours of sleep, and the like.

Cold baths are interdicted, as they throw too much work on the kidneys, but hot baths are serviceable, as they enhance skin action, and thus relieve the kidneys. They may be warm pack, Turkish, vapor or hot-water baths. Massage is particularly useful when the patient is confined to bed for any reason, aiding in elimination of effete products by the skin.

Rest in bed, when albumin is severe and persistent, or when the heart shows signs of weakness, is admirable in conserving the strength of the patient, and should be insisted upon now and then. The patient should rest in bed for ten of twenty-four hours to relieve heart action and diminish general activity.

An abundance of water is required to replace the excessive excretion of water, and to keep the solids in solution. We must not go to excess with this line of treatment, however, as the tension may be increased by ingesting too much water, and our efforts to aid would become reactive. Three liters of fluid daily is said to be a proper amount to be ingested (Anders). The carbonated waters are strongly indicated in order to counteract the high acidity of the urine which is usually present.

"An alkaline urine dissolves exudates" (Tyson).

"Malt liquors are bad; spirits are worse; the best are those which contain alcohol in from 5 to 8 per cent., such as acid wines" (Millard).

*Diet.*—Nitrogenous foods must be decreased considerably, as they increase the work of the kidney by favoring increased secretion of urea. We must not restrict the diet too much, as strength must always be maintained, and this adjustment will call for nice discrimination upon our part. Coffee and tea should be partaken of sparingly, if at all. Milk, or articles of food prepared with milk should be given an important part in the dietary. Meats and meat gravies should be greatly reduced, though fish and oysters may be freely eaten. Patients should be abstemious in dining at social functions, as the food there consumed is highly seasoned, and often washed down with the stronger alcoholic preparations.

*Systematic Treatment.*—Here our object is to inhibit or arrest the sclerotic and degenerative changes, and to overcome the ever-present and increasing anemia. For the first we have recourse to potassium iodide, hydriodic acid, and mercuric chloride; for the latter, to arsenic and iron.

Iron meets the dual requirement of an astringent and tonic, and is agreed upon by nearly all writers as being indicated, but must not be used in too large doses, as excessive doses lock up the secretions too much and cause danger of uremia.

Symptoms prominently occuring are best treated as follows:

As high tension is more or less essential in chronic nephritis with full compensation, we must carefully weigh the conditions present before we decide that the tension needs to be lowered, our guide being certain symptoms, as flushing of the face, congestive headaches, vertigo and epistaxis, while the danger of cerebral hemorrhage is always to be borne in mind.

If the heart is all right, we may use veratrum viride or aconite over long periods, while glonoin is used if any cardiac weakness is manifest.

Low tension predisposes to serous effusions (transudates), and a decrease in the daily output of urine, thus increasing the tendency to uremia, and must be combated vigorously. Here digitalis, combined with glonoin is admirable, as the heart is weakened.

Effusions may be either transudates or exudates. In either case the indication is to get rid of the effusion as quickly as possible. If a transudate, increase the tension; if an exudate, absorptives or local applications are useful. If these measures fail we may tap the cavity and inject adrenal solution, which seems to have the happy effect of preventing further accumulation of fluid in serous sacs.

Salines, cathartics, by their osmotic action, play an important part in favoring absorption.

Counterirritation is useful in the parenchymatous form, where there is so much congestion of the kidneys. Dry cups—better wet cups—are used over the kidney area, often releaving the congestion and immediately improving diuresis.

Drowsiness is to be met by decreasing and restricting the solid diet, by increase of fluids and free elimination by purging or sweating rather than by using diuretics too freely, holding this class of drugs for later use.

When edema is slight, massage, bandaging the limbs, rest in bed longer in the mornings, or a rest in the middle of the day, may be sufficient to reduce or check it. When it increases aud interferes with the function of any organ we must then rely upon diuretics and other eliminants to aid us. If the legs remain distended with fluid and the skin shining, pearly white, we may practice the "anti-philogistic touch of the therapeutic knife," as used by Pancoast, which consists in lightly puncturing the skin in many places with a fine lancet or scapel, permitting the fluid to exude from the superficial tissues. Southey's tubes may also be used when above indications are present.

Many authors speak of the dan ger of erysipelas from these procedures, but it may be avoided by asepsis and common sense.

Diaphoretics are useful from the very beginning of treatment, as their use eliminates toxins and saves the kidneys much extra work, and they should be used regularly and systematically, relying upon baths—water and hot air—with occasional use of drugs, as sweet spirits of niter and spirits of Mindererus—the latter in the well-known Basham's mixture, holding pilocarpine for occasional emergency use.

Diuretics should not be used when perfect compensation is present, but are indicated when there are scanty urine and much dropsy. When deficient heart action exists we use drugs which act on both heart and kidneys, such as caffeine, scoparius, digitalis, and Convallaria majalis.

If dropsy is due to deficient kidney function, and the heart is in good condition, we may employ the potash salts, squill, sweet niter, diuretin hexamethylenetetramine.

Asthma is a system of increasing intoxication, and is often present for a considerable period of time before other marked symptoms of toxemia develop. Elimination, particularly by the accessory organs, must be practiced thoroughly in order to overcome the condition.

Chloral may be used. At times venesection will do good. Other cases do well on small doses of morphine hypodermatically, repeated only as needed.

Edema of glottis appears suddenly, is dangerous, and needs prompt attention. Inhalation of steam, cocaine spray combined with adrenalin, scarification, counterirritation or leaching may be practiced, and, as a last resort, tracheotomy is to be done.

Epistaxis is due to high tension. The general indication is to lower blood tension. Locally, pressure of the non-elastic capillaries will do good.

Obstinate vomiting or diarrhea is a symptom of great toxemia, and must be promptly met. Vomiting is usually relieved by lavage; diarrhea by sweating, or diuretics. Do not check the diarrhea completely, as it will cause retention of toxins, and may precipitate uremia.

Pruritis is usually due to urea crystals irritating the skin. Hot bathing, followed by 4-per-cent. carbolic acid solution, infusion of conium leaves, or chloral, may be useful.

Palpitation is met by the use of Convallaria majalis, and, at times, morphine.

If the nephritis is dependent upon other diseases as a causal factor, the primal disease is to be treated, and the condition of the blood pressure and urinary excretion are our guide to the treatment of the nephritis.

Uremia must be discussed, as the symptoms of chronic nephritis are often really those of mild degree minus convulsions.

When convulsions, unconsciousness, etc., appear, we know that failure of compensation has occurred, and that this point marks the beginning of the end.

Venesection should be done at once, whether tension is high or low, as it speedily eliminates toxins. This is to be followed by injection of normal saline solution, dilutes the remaining portion of the blood and stimulates the various functions, guarding against raising the blood tension too much.

Spinal puncture has been resorted to recently upon hypothesis that uremia is due to edema of the brain, but the results have not been conclusive thus far.

For the convulsions occurring in the parenchymatous form, morphine in good doses may be used, but in the interstitial form may be used sparingly. Chloroform, chloral, and the bromides are also used to combat the convulsions.

The matter of using morphine in the uremia of chronic interstitial nephritis has been largely discussed, many authorities being found who take sides on the subject. While on duty under Dr. D. E. Hughes, at the Philadelphia Hospital some years ago, the author saw him use it in many cases of uremic convulsions, and can not recall one case where any ill effects were noted.

Personally he treated one case of chronic interstitial nephritis for over five years, during which time a pronounced broncho-pneumonia and three attacks of uremia developed, using morphine hypodermatically for asthma that resisted other measures to combat, always with happy results. His usual routine was to give pilocarpine the morning following the hypodermic, and he never saw any untoward effects of its use in this case.

Exacerbations are to be treated exactly the same as the ordinary acute attacks.

Edebohls' operation of stripping the kidney capsule in chronic nephritis seems to do good in nearly every case selected by him, and other careful operators, and is simply mentioned here, as it is out of our domain in the limits of this paper.—*Merck's Archive.*

---

### NEW PROBLEMS IN THE TREATMENT OF MALARIA.

The recent truths developed in the production of malaria have forced many new problems for the medical man and health officer to work out. We now know that protection by mosquito destruction consists in the draining of swamps, the filling up of stagnant pools, the closing of tanks near houses, the clearing of undergrowth and close vegetation together with the liberal use of kerosene oil on these various breeding-places. Unfortunately, however, experiences show that this plan while scientifically sound is very difficult of application in actual practice, for the drainage of the waste parts of the earth is a vast problem.

We now know that the mosquito can not fly far and can not retain its power of propagating malaria unless it finds shelter within one hundred and fifty yards of a habitation; hence the destruction of breeding-places within the area in which any dwelling stands to this extent should free the place from malaria, but as all malarial-bearing mosquitoes do not bite only at night and as during the day the inhabitant must be abroad, beyond the "healthy area" he becomes infected in this manner.

As a protection against mosquitoes the use of fine gauze around the bed has been recommended but gauze is apt to tear, to get foul and smell "earthy," and unless its meshes are very fine mosquitoes will get through it; wire netting has come into use lately, and possesses many advantages, but the disadvantages of any kind of netting around the bed is that the air within it grows very close and stagnant, rendering sleep impossible in very hot weather. Moreover mosquitoes bite in the early evening, and by biting the ankles through the stockings, the head or the hands, can inoculate the human being, so a mosquito-protected house is theoretically the ideal form of warding off mosquitoes.

Again the local distribution of some of the anopheles species is peculiar, showing some important differences in the propagation of malaria, for although several varieties of anopheles may be present in a locality, it by no means follows that all or the majority convey malaria to man; in fact, only a few are capable of transmitting malaria. Mosquitoes although incapable of existing in numbers away from plentiful vegetation, may be carried from a place where they prevail in numbers to districts where they are unknown, in the clothing, baggage, etc., of passengers, and in goods sent by railway; they were transported from Rome to London and still possessed their power of infecting. Manson and Warren, of the London School of Topical Medicine, were infected by malaria-infected mosquitoes from Italy. But the theory that malaria is carried long distances by prevailing winds is probably incorrect, as mosquitoes in strong winds seek shelter. Malaria can appear as a new disease in a place, or in the form of a recrudescent outbreak, as illustrated in the case of the island of Mauritius, and in the town of Middleburg in Zealand, Holland. In Mauritius, malaria was unknown until about the year 1876, when it broke out in a very severe form; while in Zealand, according to Van der Scheer and Van Berlekon, malaria reappeared there after a lapse of thirty years. That mosquitoes infect human beings with malaria as far north as Holland enlarges our views as to the conditions of temperature, etc., under which malaria and malaria-infecting mosquitoes can maintain their power.

Malarial parasites in the blood without fever have been frequently noted in the case of children in tropical climates; this is explained by some observers as being due to an immunity transmitted from the parent to the offspring; a theory which is wholly unproven as yet, for experiments go to show that natives do not suffer less than Europeans from mosquito bites. The irritation caused by the bites is less in the case of the native, but mosquitoes are attracted, at least in Sierra Leone, more by the native than by the Caucasian; as was shown by noticing that in a tent in which a European slept only a few anopheles were caught, whereas in the same tent anopheles swarmed when natives slept in it. After the departure of the natives anopheles again diminished in numbers. It would appear rather that the immunity of the adult natives is due to the fact that in childhood they all suffered continuously from the disease.

As to new remedies in malaria, the bihydrobromate of quinine, which dissolves readily in six parts of warm water, has been administered, in malaria, hypodermically; injected in 3-grain doses subcutaneously, dissolved in 20 minims warm water; six injections, on alternate days, sufficed to cure. This drug should be tried in regions where the malaria is severe. White usually injects 5 grains of the bisulphate of quinine repeated every three days, selecting the subcutaneous tissues at the angle of the scapula, for two or three injections which generally suffice, while Powell employs the hydrochloro-sulphate of quinine in 18 or even 36 grains at one injection; one part of the salt being dissolved in 3 or 4 parts of a 1 in 5000 solution of perchloride of mercury. Dowler uses (1) the ordinary sulphate of quinine dissolved in tartaric acid; (2) the neutral sulphate of quinine; but he prefers (3) the bihydrochloride of quinine as being more nearly painless.

Gray uses intravenous injection of ¼ to ½ of 1 per cent. as quinine in decinormal salt solution; he prefers 30 grains of bimuriate of quinine and iron in a pint of decinormal salt solution, injected into a vein or into the subcutaneous tissues, and if done slowly with antiseptic precautions he states that it is not a serious operation.

Benakz prefers the use of large doses of arseniate of quinine—18 grains daily in the form of cachets or pills, in the treatment of malarial diseases, for he regards the arsenic present in the salt as a negligible quantity, and innocuous as far as its therapeutic specificity is concerned. Arseniate of quinine acts both as a febrifuge and as antiperiodic; no deleterious sequalae accompanied its administration; and it has produced in Benakz's practice excellent results in the various forms of malarial diseases. Methylene blue Iwanoff and Wood, Jr., find acts chiefly on the protoplasm of the tertian parasite, whereas quinine affects the pigment. By administering 5 grains thrice daily these authorities found that in the tertian parasite the changes began at the end of the second day, and affected only the fullgrown forms of the parasite, whereas the younger forms remained unchanged. Quinine acts in quite the reverse way, for it is the younger forms that are chiefly attacked by the drug, and the adult forms scarcely at all, so this drug is indicated in cases where quinine fails.—*Med. Times.*

---

### SOME REMARKS UPON THE USE OF THE UTERINE CURETTE.*

BY J. WESLEY BOVEE, M.D., WASHINGTON, D.C.

Professor of Gynecology in the George Washington University; President of the Washington Gynecological and Obstetrical Society, etc.

For a considerable number of years I have used the uterine curette for many different conditions. By this fairly large experience I have developed the curette touch, to use an expressive though unusual phrase, that the gynecologist should have and usually has. Three styles of the instrument, viz., the dull, the sharp and the serrated, which latter may be either dull or sharp, are in use. These are not to be employed indiscriminately. The dull variety is to be used for the removal of shreds of tissue after abortion or miscarriage. Even after full-term labor the use of this instrument becomes necessary at times for practically the same indications. The sharp serrated instrument is employed in the removal of intrauterine growths. We are all familiar with the serrated spoon curette of the late T. Gaillard Thomas, of New

*Read before the first meeting of the Medical Society of Northern Virginia, at Alexandria, Va., May 17, 1905.

York, devised especially for this purpose. The plain sharp curette is the one most frequently employed as the indications for its application are more varied and commoner. In all forms of endometritis not associated with malignant disease of the uterus that does not spring from the mucosa, carcinoma of the body, pediculated submucous fibroids, mucous polypi, subinvolution, metritis and chronic passive uterine congestion the sharp, plain instrument may be employed. In suspected carcinoma of either the cervix or the body curettage is done for the purpose of securing tissue for microscopical examination. In such work there is danger of allowing too much time to intervene between the curettage and the radical operation should a microscopical diagnosis of cancer be made. The absorbents are stimulated by curettage and the disease may be quickly carried to unremovable structures even when unsuspected. Pryor and others have stated not more than ten days should elapse. I believe I have seen this effect shown in three days. In advanced cancer of this organ thorough curettage and cauterization is the classical treatment. In endometritis very often the accompanying uterine retro-displacement that creates an intrauterine reservoir is the principal condition requiring rectification. The dull serrated curette is applicable to practically all the conditions for which the plain dull curette is used and is by some considered preferable. These are practically all the conditions in which uterine curettage is applicable. These might be termed the legitimate uses of the curette.

The abuse of this instrument deserves our very earnest consideration. Its employment in the endometritis accompanying large uterine fibroids that encroach upon the uterine cavity is, in my judgment, reprehensible for the following reasons: First, it is impossible to reach the pocket or pouches above the growths and in which the endometritis is intensest. Any one who has split many fibroid uteri must have been impressed by the unapproachable pockets and the state of the endometrium lining them as contrasted with the remainder or the uterine lining. Second, the danger of infection is an element that must be considered, as very often infectious material lurks in these fossæ and denudations invite its absorption. Third, the tendency to hemorrhage in such conditions, when disturbed by the curette, is far from being a fancy. Fourth, the preparations of the patient and the necessary administration of general anesthesia are practically the same as for more radical procedure. This last should be borne in mind whenever this operation is considered as an alternative for any of the more radical operations upon this organ. When fibromata have undergone malignant degeneration or are accompanied by such condition in other portions of the organ or have become infected, the use of the curette becomes not only interdicted but absolutely harmful, except when extirpation of the malignant growth becomes impossible. In sarcoma of the uterus in stages not too advanced for extirpation curettage should never be done.

The employment of the sharp curette in puerperal infection—a practice altogether too common, is reprehensible, as it permits rapid absorption of the remaining septic material, the removal of which can hardly be expected. It is believed obstetricians are gradually realizing this contraindication of the use of the sharp curette. In the endometritis accompanying backward displacements of the uterus curettage can be of little service except the proper position of the uterus be subsequently maintained. When this latter precaution is overlooked the patient is placed on her back in bed directly after the curette has been used and the retrodisplaced uterus is thus hopelessly deprived of drainage—a factor of the greatest importance at this time. I have repeatedly relieved patients of uterine hemorrhage and offensive leucorrhea following, repeated curettage, by careful exploration of the uterine cavity and the removal from it of small growths such as fibroids or mucous polypi. I may also here refer to a practice of some physicians of doing curettage in their offices and sending the patient home afterward. This I consider malpractice in as much as the technique of such procedure must be very faulty and the danger of infection enormously enhanced. Repeatedly I have seen pelvic peritonitis, usually suppurative in character, follow such procedure and am confident a large number of women have had their tubes removed because of pus tubes created in this manner. I should like to protest against routine curettage being employed when plastic work on the cervix or the perineum is done. I believe this is a quite common practice.

A few remarks upon the technique and after-treatment of curettage patients may not be out of place here. I never enter the uterine cavity except under a very careful technique without great trepidation. Even the introduction of a uterine sound without such care is limited in my work to perhaps two or three instances annually and necessity for it is regretted. We should remember it is a surgical operation and done upon an organ practically the most important in womanhood and one in which infe ction so easily created may lead to a fatal termination. The vulva should be carefully shave d and prepared and after curettage the uterus carefully swabbed with small pieces of sterile gauze that the removal of all debris may be assured. In the after-treatment antiseptic vaginal douches should be employed one or more times daily for eight to fourteen or more days. The patients should be kept in bed from eight to fifteen days after operation and at the following one or two menstrual periods. The bowels should not be allowed to be become overloaded. Exercise of these patients during the following two months should be very limited.—*Virginia Medical Semi-Monthly.*

---

### Boils.*

By George W. Guthrie, M.D., Wilkesbarre.

It may seem that some sort of apology were due from me for presuming to occupy your time with a subject so trite and commonplace as the above, still it may be known to some of my confreres that when this honorable body met a year ago at York, I was the unwilling host of several of these "comforters," affecting my right hand, due to infection acquired a short time before the meeting.

The boil has been the subject of idle jest, of funny hit and happy fling; but there is a serious side to the subject and one possessing a deal of scientific interest.

Leeuwenhoek, over two hundred years ago, demonstrated by his

*Read at the meeting of the Medical Society of the State of Pennsylvania, held in Pittsburg, Sept. 27-29, 1904.

microscope that the dust of the moth miller's wing is not an amorphous substance, but one composed of beautiful feathered shapes like the plumage of the higher-winged creatures. So the most unsightly pimple that mars the face of beauty or adorns the features of the schoolboy embodies in its growth and development all the processes of infective inflammation.

Samuel Rogers says:

"That very law which moulds a tear
And bids it trickle from its source;
That law preserves the earth a sphere
And guides the planets in their course."

And following this method of comparison, we find the same laws and rules to govern the growth and development of the boil or furuncle as those governing the most serious forms of infective inflammation.

Pustules, boils, or furuncles and carbuncles, are all classed under the same head; namely, suppurative diseases of the skin. These affections are due to the invasion of hair follicles or sweat glands, and sebaceous glands by the various pyogenic cocci. Garré demonstrated this by rubbing pus into the sound skin of his forearms and obtaining a crop of furuncles. Some three or four years ago I had under treatment a very obstinate case of pemphigus, the body studded with the growths in various stages of suppuration. An eminent specialist in dermatology was called, and advised a continuous bath by keeping the patient immersed in tepid water. This was done. Within twenty-four hours the temperature rose to 104 degrees, delirium was wild, and the patient succumbed. The nurse, a model of faithfulness, trained in the English Army Hospital service, had his hands and forearms constantly under water for hours, endeavoring to control his delirious patient. In two or three days after the patient's death, the nurse's arms, forearms, wrists and hands became the seat of a very numerous crop of furuncles.

Physicians and nurses whose duties call them to care for pus cases are very prone to this kind of infection. But in this, as in other infectious diseases, the invading bacterium is only one of the causes. There must be a suitable soil, and this is produced by a lowered vitality or diminished resisting power in the patient. A doctor or a nurse, tired and worn by prolonged vigils and insufficient rest, is rendered susceptible to this and other forms of infection. There are, also, certain diseases, as diabetes and nephritis, that predispose to this trouble. Certain portions of the body are favorite sites for these troubles: the outer aspects of the back of the hands, the back of the neck, and the nates. This is probably due, in a great measure, to the growth of hair in these places; also to enlarged sebaceous glands and hair follicles, and to want of cleanliness, especially at the back of the neck and the nates. Slight abrasions, made by scratching with dirty fingernails or the edge of a collar, afford avenues for the entrance of the specific germ.

As claimed above, all the steps of infective inflammation are here illustrated. Here is the specific germ, the pus-making cocci invading the follicle as an irritant; then we have congestion and stasis, and exudation and the termination either in resolution or in suppuration, usually the latter.

Clinically, the first indication of a boil is a slight elevation surrounding a hair follicle with some burning or itching; this soon becomes pustular and we have the primary pimple. If this be let alone and some protection thrown around the elevation, it may subside and abort. But if this slight pustule be broken and squeezing be resorted to, the trouble is greatly aggravated; without this interference, the inflammatory swelling is apt to increase and a cone-like elevation appear. In a few days a slight breaking down in the center takes place and a small crater-like opening appears at the summit; usually a fine probe may now be inserted into the opening to the depth of a half-inch or more.

In the case of carbuncles there are several hair follicles involved; a colony of furuncles and the inflammatory action extends to the subcutaneous cellular tissue; without interference the process of breaking down in the center continues; the crater becomes larger and larger, and, finally, a slough or core separates and the remaining process consists in the healing of the resulting cavity.

The treatment of this class of trouble consists of preventive, abortive and curative treatment.

In the way of preventive treatment measures should be used to restore the normal condition of the patient; if tired and overworked, rest and recreation with plenty of fresh air and good food; in fact, anything to restore the normal vitality.

Care of the skin is important—proper bathing is indicated. Slight injuries of the skin should be avoided, and when they do occur they should be treated by covering them so as to prevent the ingress of the pyogenic cocci.

In the way of medicine, some authorities have lauded the sulphite of calcium. I have used it quite extensively without any apparent results for good. In my hands quinin in moderate doses has done good service. Fordyce Barker says, "I know of no remedy so effectual as a preventive of suppuration as quinin." It has been my practice to give three grains of quinin three times a day for a week, and with excellent results.

*Abortive Treatment.* Can anything be done locally to abort these troubles? My preceptor in medicine, Dr. Edward R. Mayer, believed that a saturated solution of permanganate of potassium applied freely and early was very effective. Others believed that a minute drop of pure carbolic acid insinuated into the hair follicle will produce the same results.

The injection of carbolic acid in various degrees of strength has had its advocates, as has also the crucial incision and the application of carbolic acid, or what Agnew calls a "twofold barbarism."

Practically, I am a disbeliever in the abortive treatment or, rather, I believe that more harm is done than good by this form of treatment. An intelligent, practical layman, a kinsman of mine, has often told me that pricking the primary pustule and squeezing the swelling was invariably followed by aggravated symptoms.

In this, as in many things, it is remarkable how laymen have forecast and recognized the sound principles of medical science. The old grannies, when they scorched the linen for dressing the umbilical cord, sterilized the dressing, although they did not know it; the old nurses when they scalded the milk for sick babies applied the most approved principles of sterilization, although they were ignorant of the reason; the mother who tied her boy's cut finger "up in the blood," kept the wound bathed in the sterile blood and secured healing *per primam* without a change in dressing. They were all applying the principles of sterilization, though they were ignorant of the reason why.

So in the case under consideration: Pricking the slight pustule and squeezing the cone-like little boil forced the infective agent around which nature had thrown a wall of protection into the surrounding tissues and increased the area of inflammation. This principle is now recognized by the profession.

Lilienthal says that in shaving the hair in the neighborhood of boil, the razor should be drawn from the base of the apex so as not to drive the infective agents deeper into the tissues.

Warren says that, in syringing out the crater-like cavity attending the earlier stage of the boil, "care should be taken *not* to overdistend the pus cavity or the septic process may be made to spread and all the symptoms be aggravated." For the same reason, I consider it unwise in the early stages to incise and cauterize.

A year ago, during my attack referred to above, an enthusiastic medical brother who took a very pessimistic view of the trouble, advised and urged incision, and cauterization with pure carbolic acid. I consented and submitted to the "twofold barbarism" in the case of one of the swellings. The result was that the treatment was immediately followed by marked aggravation of all the symptoms. The hand became greatly swollen; the lymphatics of the forearm tender and the supratrochlear gland on that side much swollen, while the other boils on the hand that were treated by a "masterly course of inactivity," gradually aborted and became innocuous.

Now a word as to the curative treatment and I'm done. The old-fashioned flaxseed and bread and milk poultice does harm rather than good, by softening and preparing the soil for the reception of the infection.

The so-called antiseptic poultice, as gauze saturated with a boric acid solution, or with Thiersch's solution, is an admirable dressing. As soon as the crater-like opening at the summit of the cone will admit a probe or small grooved director, and there is evidence of pus beneath, a small straight bistoury may be inserted and the opening enlarged so as to permit a free discharge of any retained pus. This will give great relief to the patient. Then day by day, the softened slough, or core, may be gently pressed out by using cotton sponges saturated in boric acid solution or a weak bichlorid solution; and when the cavity is cleansed, healing will take place very promptly. No free incisions, no curetting or injections into the cavity will render any more satisfactory service, but aggravate and prolong rather than shorten and relieve the trouble.—*Pennsylvania Medical Journal.*

---

### PHIMOSIS.

This is a condition every physician has to deal with. The obstetrician and pediatrist have to deal with the congenital form and the general practician has to do with all forms. In phimosis we have not simply a redundancy of preputial tissue, but with it, and in some cases without it, a constriction of the mucous membrane as at its orifice so that the prepuce can not be retracted over the glans penis. In some cases there is simply a pin-hole opening and in others the glans can only be partly exposed to view. Whenever an unusually elongated prepuce can be readily pushed back of the corona glandis and no tightness exists, then we do not have phimosis but simply an excess of preputial tissue.

We have two varieties of phimosis, the congenital and the acquired, and in either one the prepuce may be excessive, normal or deficient. The congenital variety may be divided into the simple and adherent forms. A few of the congenital forms *may* correct themselves, but they are nearly aways permanent. The acquired variety may be divided into the inflammatory and cicatricial forms; the former is often only temporary while the latter is always permanent. Some of the pronounced acquired cases have already existed mildly in the congenital form.

In intra-uterine life, when the preputial tissue grows forward its inner surface becomes adherent to the glans and, as a rule, during the first year after birth these adhesions become loosened; if not, then they become permanent if not separated by the physician.

The mucous lining is not as elastic as the integument, and if the opening of the prepuce is small at the time of birth retraction is often impossible. Heredity is supposed to be a cause. In the acquired forms the etiology consists chiefly of accidental conditions, as trauma, inflammations, edema, gonorrhea, syphilis, ulcerations and bad circumcisions.

On account of the inflammation, tumefaction and other forms of narrowing of the preputial orifice there is an accumulation and retention of smegma, subpreputial secretions and urine. Decomposition and other changes come about and in some cases formations of calculi of the phosphate of lime and ammonio-magnesium phosphate result. These retained substances cause irritation and numerous local and reflex symptoms.

Locally, there may be an itching or pain of the head of the penis, and pruritus ani may accompany it. Urination is usually frequent and painful; sometimes both nocturnal and diurnal enuresis exists; occasionally blood has been noticed; there may be incomplete emptying of the bladder, or even retention of urine. There is sometimes a sudden stopping in urination, resembling stone in the bladder. Straining at urination is a very common condition and is the cause of many cases of prolapsus ani and of hernia—umbilical, inguinal and scrotal. The child often cries while passing water or may cry out at night while asleep. The parts are unclean and irritated; the boy patients pull at or titillate the penis; priapism is sometimes present (especially in older ones), early sexual excitement results, and the habit of masturbation is indulged in and established. Later, spermatorrhea and nocturnal emissions are common. Later in life, sexual intercourse may be without pleasure, difficult, painful or impossible. We have irritation, inflammation, edema and, in some instances, cicatrices resulting. When simple posthitis is present the prepuce pouts, is swollen, red or purplish, tender and doughy to the touch. Balanitis is frequent. Urethritis is sometimes present and cystitis is possible by extension, at least the bladder is very frequently irritable reflexly. Ulcerative balano-posthitis occurs with all the symptoms of inflammation and a foul, purulent discharge. The inflammation may become chancroid in nature when the prepuce may ulcerate through so that the glans penis can be seen.

The reflex conditions are serious in many instances. The patient has various symptoms, becomes irritable and discontented, peevish, mentally dull, forgetful, sleep and appetite are disturbed, nutrition is imperfect in some, and the general health may become permanently impaired. Convulsions are frequent, chorea occurs and so do reflex peripheral paralysis. Various incoordinate muscular movements appear reflexly, especially of the lower extremities. Paralytic strabismus has been produced by phimosis. Vasomotor and trophic changes are common. And finally, phimosis, through masturbation, may be the cause of epilepsy.

Although a moderate phimosis may be without symptoms, yet it predisposes to trouble from injuries and to infection from coitus with unclean women.

As complications we find shortness of the frenum, narrow urethral orifice, adhesions, fissures, herpes, vegetations and some claim that late in life the condition strongly predisposes to cancer of the penis. Paraphimosis is a frequent accident.

Since any one or several of these symptoms are probable during the lifetime of a male child, it can well be understood that attention should be given to every penis at the time of birth to see that the foreskin can be pushed back of the head, the smegma removed and the parts kept clean. When the phimosis is not marked, daily retractions and cleanliness may cure. When slight adhesions prevail, a small blunt probe passed between the preputial covering and the glans will correct the condition.

Forcible tearing of the mucous membrane only has been recommended by some, but this leaves a troublesome and painful condition to attend to or bad adhesions will form, or even if this is prevented the possibilities of complications later in life are not done away with. Do not resort to this.

Slitting of the prepuce along the dorsum of the glans has been frequently done, but this is usually an unsatisfactory operation and the parts remain in an unsightly condition. It is justifiable only in cases where the preputial tissue is normal or deficient in length.

Unless the child has a very bad heart or is of a family of "bleeders," the operation of circumcision can be done in all male children with benefit, as is proven by the Hebrew people. In normal or very mild cases it may be diplomatic not to urge an operation, but whenever there is a pronounced narrowing of the preputial orifice and great redundancy of the tissue, no exposure of the glans is possible, adhesions are firm, inflammations exist or the reflex symptoms have appeared, then there is but one duty for a physician and that is to advise, to obtain the consent, and to do, a *circumcision.*

In children the following method is the one we recommend : Grasp the foreskin with a couple of forceps and bring it forward under fair tension, then apply the phimosis forceps transversely immediately in front of the glans penis and with a sharp knife or a pair ot small scissors sever the tissues through the fenestrum of the instrument. When the prepuce is completely severed, the integument will retract but the preputial mucous membrane still covers the glans. A grooved director is then passed under this and with a pair of scissors a slit is made along the dorsum to the corona. Two triangular flaps of mucous membrane now exist and each one is trimmed so as to leave enough to pass the sutures and unite it to the skin. Four sutures are usually sufficient to bring the edges nicely together.

In adults the operator may, or may not, use the phimosis forceps. Great care must be taken or too much of the skin layer will be cut off—a very undesirable mishap. On the other hand, not too much (not over one-fourth inch) mucous membrane must be left, or the covering may slip in front of the corona and a secondary phimosis occur if the mucous membrane contracts.

Local anesthesia is employed by some, but we prefer general anesthesia. Antisepsis before any cutting is done, after this asepsis. Cat-gut or silk sutures can be used according to the choice of the operator. Many kinds of dressings are recommended, but plain *sterile* vaseline on *sterile* gauze is ideal and can be readily applied and removed.

When a *circumcision* is carefully and properly done, good and permanent relief from all symptoms will result, while the other methods of treatment can not be relied upon.—*Medical Council.*

### THE SEXUAL NECESSITY.*

BY E. L. KEYES, M.D., LL.D., NEW YORK.

I have been allotted ten minutes and asked to present briefly a medical point of view, a suggestion as to the lines along which this Society shall approach the community in an educational way—for this is an educational symposium. And it seems to me that I may do this best by attacking the matter at its kernel.

The gist of the whole subject, as I believe, lies in the sexual problem, a problem we usually discuss behind closed doors; but the doors must be opened now if the Society expects to accomplish anything educational. The dissemination of knowledge along pathological lines will doubtless be helpful in deterring the stupid, the ignorant but innocent and right-minded from spreading disease of such obvious and widespread errors as a belief that the danger of communicating syphillis ceases upon the cicatrization of the chancre; the old, oft-quoted fallacy that a gonorrhea is no worse than a cold, the belief that a mild gleet is no longer contagious; and, finally, the most abominable of fallacies that a proper treatment for a mild gleet is sexual intercourse—even matrimony.

With a broader knowledge upon all of these points is most essential to the community in its own interest, yet this does not hit the root of the evil which, in my opinion, is the dual standard of sexual morality for the two sexes, based largely in modern days upon the idea that sexual exercise is, on the whole, sanitary to the male, if not essential to the best performance of his other general physical functions and necessary for the preservation of his sexual potency.

This idea, upheld by a considerable minority at least, perhaps by a majority of the medical profession, as well as by a large contingent of the laity in a loose way—this idea, I repeat, is the nucleus of the whole matter—and it is this idea that I propose to discuss.

From a medical source should come some autboritative utterance in controversion of this fallacy upon which, as a foundation, the lay

* Read before the American Society of Sanitary and Moral Prophylaxis, April 1905. Reprinted from the Medical News, July 8, 1905.

educator may build—amplifying and elaborating his superstructure to any extent.

The early man probably obtained his wife by physical prowess and held her in subserviency, he doubtless doing the fighting and the bread-winning and she ministering to all his needs and wants, more as a favorable servant than anything else. He was prince and she was subject to him. The right of man to exact sexual tribute from the weaker sex appears to have been granted as a matter of course. All the histories of prostitution touch upon this subject and make it clear that, in the earliest days in the East, it was considered a part of the duty of host to provide for the sexual necessities of those to whom he owed the rite of hospitality, even considering it a courtesy to go so far as to offer his daughter for the accommodation of his guest. And this custom in its entirety, I have been told by a guide conversant with the remote regions of Morocco, still obtains in that district to-day.

Man in his strength and physical might, therefore, exercised a power of selection over the weaker sex and appropriated or dispensed the favors of the female at will.

The fighter seems naturally to be sexually overvigorous; the continuous physical worker and the athlete in training not so.

I am told by a general in the United States Army, who has seen a large amount of campaigning among the Indians in our Northwestern country, that, in his experience, the Indian undergoes a period of rut in the spring-time. Then it is that he paints his face, bedecks himself with feathers, and goes upon the warpath; and, when a band has raided a settlement—while the fighting is still going on—the warrior rushes in among the burning tents of the village of the enemy, and at once proceeds to ravage the women.

Women, as a rule, admire a fighter; one who is bold and aggressive appeals at once to them and is granted a favorable reception in a general way, while the feeble, effeminate male is looked down upon and turned slightingly aside.

Moreover, it is the well-known habit of the community to look leniently upon the sexual errors of the male, while those of the female are frowned down upon with unrelenting severity—as if to say, the lily once smirched is ruined forever, while the coarser fiber of the oak leaf may receive many a bruise and still be accepted by the community. This is illustrated forcibly by the popular sentiment existing to-day in the South. If a black man is known to have sexual relations with a white woman he is run out of the town, and fortunate if he escapes lynching But if a black woman seduces a white boy, the matter does not appear to be so serious—lynching the woman, at least, is not one of the sequences.

In the same way, any jury in the North will deal gently with the man who shoots at sight the villain who has seduced his wife or daughter; but no one thinks of shooting the woman who, by her wiles, has seduced his son.

This spirit of recognizing a certain sexual right on the part of the male, therefore, still exists and is accepted and tolerated by the community as a whole—not by the church, not by the law, not theoretically by the community—but practically it is a fact that it is so tolerated.

How, then, is this sentiment, this tradition, this unwritten law to be overcome? Only by educating the young male, teaching him to develop his character, teaching him that his sexual yearnings are to be restrained like his propensity to over-eat, to over-drink, to over-exercise; teaching him that because he wants a thing it is not, therefore, necessarily good for him that he should have it, but often harmful; by assuring him that not only will he not perfect or retain his sexual potency by indulging in illicit intercourse, but that he is more likely by excess and irregularity to impair it—and this information it is the province of the medical profession to impart, and to impart it forcibly, speaking as a unit and with no uncertain voice.

But how shall we prove this last proposition upon which there are held diametrically opposite opinions in the profession? There is not time nor would there be much purpose in appealing to many authorities. I shall cite two, however, as types.

Milton, in his work on gonorrhea, boldly states that sexual intercourse is necessary for the preservation of the sexual function. He takes the broad stand that any organ, not exercised, gradually but necessarily loses its power, and that impotence is the direct consequence of continuance—of course citing cases.

Acton, "On the Reproductive Organs," on the other hand, with equal insistence claims that absolute continence is compatible with perfect preservation of the sexual function and, equally, cites striking cases in proof—and so on through the list of authors.

Is this expression of opinion then a matter of personal point of view? I think, manifestly, yes.

Any one reading Milton's book is at once struck by the force, the vigor, the rankness of his style. Here is a physical man rejoicing in the air he breathes, the food he eats, in every vital act. He was, doubtless, full of physical necessity and recognized, as many stalwart young men certainly do, a consciousness of physical comfort a clearing out of the cobwebs of his brain, a sense of *bein-etre*, when following a regular and systematic sexual life, that was absent when his sexual relations were interrupted. There are many, very many, such individuals in the community; and if they can not marry, they habitually resort to illicit connection and, because they feel better after the act, persuade themselves that they can not live without it; and this is particularly true if they allow their imaginations to have full play.

Read Acton's book, on the other hand. Here is an intellectual type. He is no gross feeder and is not in any sense carnally-minded. His delights are in the pleasures of the mind and the soul—and yet he is forceful, shows character, and is surely a manly man.

So one gets a few cases that support his thesis and generalizes from them; and the other does the same. Which is correct?

But now, leaving generalities, let me resort to the direct appeal.

One of you, gentleman, has a charming daughter. She is delicate and refined, sensitive, emotional, but pure in heart and has no thought or suspicion of any sexual appetite, desire or necessity. She has reached the age of—let us say thirty years, and you recognize that slowly, but surely, her virginity is crystalizing and that she is getting angular in mind and body; in short, that she is becoming an old maid. She does not know it; but you do. She is suffering from balked maternity.

Now, put yourself the question; you who, in case of a young man apparently suffering from distended seminal vesicles or the teasing of insistent sexual desire, are perhaps willing to suggest sexual intercourse—would you brutally say to this fair sufferer—go and get a man? God forbid! Rather let her suffer; rather let her die, if need be, than debase herself and sully her vestal purity.

And why should not the same standard be held up to the boy? Why should not he be helped to form character by resistance and to cultivate manhood by honest purity of mind and body? For it is the imagination that does the mischief. Except in case of disease or strong inherited tendency, the sexual appetite is not insuperable. Very many honest citizens reach manhood without ever having practiced sexual intercourse and are none the worse for it. Most of these have had nocturnal emissions and very many, it must be admitted, have practiced self-abuse more or less; but the braver and purer minded among them are not injured by these occasional lapses from integrity and, if their minds are kept reasonably clean by manly exercises and outdoor sports, they are not in the least degree menaced by impotency. On the contrary, impotence, nervous or real, is vastly more often the result of sexual excess than of sexual continence, as the case-books of any practitioner interested in genito-urinary work will attest.

The testicle is a gland like the tear gland, largely under the control of emotional influences. It is not concerned in the ordinary actions and doings of everyday life. That one, by practicing the emotion of grief or sadness and weeping copiously once a week or oftener may learn to perform the act of crying more creditably than some harder-hearted individual, I will admit; but this individual would not lose the power to cry even if he did not exercise it for half a century.

And finally, to leave the theory and opinion and come down to a matter of hard fact, capable of physical demonstration: It may be safely and surely affirmed that no amount of continence ever caused atrophy of the testicle.

Now, if continence, too long continued, can produce impotence, that fact should be evidenced by a wasting of the testicle. And I confidently assert that this is never the case; nor has any one, to my knowledge, ever claimed it to be so. On the contrary, atrophy of the testicle, to a greater or less extent, very often follows prolonged sexual abuse and excesses—notably those of masturbation, because the latter are more excessive. Indeed, throwing the testicle wholly out of function forever in a physical way—as occurs after the blocking up of the excretory duct on both sides in double gonorrheal epididymitis—does not produce atrophy of the gland, as all physicians know perfectly well to be the case.

If there is anything more pitiable in life than the young sexual neurasthenic, debilitated by sexual irregularity and excess, it is the old man, the victim of years of irregular and illicit indulgence, whose mind still gloats over forbidden pleasures which his body can no longer gratify. Death is his best friend to terminate an unhappy and poluted existence.

If the youth, sowing the wind, could appreciate fully the whirlwind he is likely to harvest, it would stay him in many a mad act and conduce to the salvation of his body as well as of his soul.—*Louisville Journal.*

---

### PERSONAL EXPERIENCE IN THE EMPLOYMENT OF MECHANICAL VIBRATION IN THE TREATMENT OF RECTAL DISEASES.

BY WILLIAM L. DICKINSON, A.M., M.D.,
SAGINAW, MICH.

*Mr. President and Fellows:*

It is only since our last meeting that I have been employing mechanical vibratory stimulation in the treatment of rectal diseases. While at Atlantic City, Dr. Evans called my attention to the benefit derived from its use in giving immediate relief to inflamed and protruding hemorrhoids. This set me to thinking, and upon returning home, I procured Kirke's Handbook of Physiology, and Pilgrim's Vibratory Stimulation, and went to work to learn why and when one should employ this, to me, new physiological treatment in proctology. Having learned a few of the great number of facts to be acquired before one can expect to treat his patients intelligently, I had a machine installed in my office and began using it, as I thought, *secundem artom.*

My first case was an irritable anal ulcer of one year's duration, and had the patient consented, I should have incised the ulcer, end to end, and trimmed away the indurated tissue, getting, in all probability, a good result, but, debarred from this trivial operation, recourse was had to the vibrator. The hard rubber ball vibratode was employed, over the first, second, third and fourth lumbar nerves, as these nerves control the anal sphincters. Pressure was made on each nerve for about twelve seconds, vibrating each one twice, a short stroke with four thousand blows per minute being used. In order to obtain the nerve endings in the ulcer locally, this small vibratode was lubricated with vaseline, and while in motion (oscillating) it was gently and carefully introduced into the rectum.

There was a little discomfort at first, but in a few seconds the patient said the treatment was painless. The duration of the treatment was three minutes and were given daily for five days, and then every second day for one week. There was applied to the anal verge, between the vibratory treatments, a five per cent. ichthyol ointment. The patient was free from all pain after the second treatment. Of course, I was greatly pleased with the result obtained, and the three following cases yielded just as readily to vibratory stimulation; but my fourth was a very nervous person, afraid of the treatments, and objected to the noise of the motor. Six treatments were given as outlined, and not benefiting her in the least, a solution of beta-eucaine was injected, the fissure incised, and in a few days the patient was well.

In tight anal sphincters where dilation is necessary, it can be done by using this vibratode, employing five thousand short strokes per minute, gradually introducing the instrument. It has been my custom to take about one minute to introduce the vibratode to its full size, hold it in place half a minute, then gradually withdraw it, repeating this procedure three or four times, when the anal sphincters will be relaxed.

I have found this the preferable method to-day for gradual dilation of the anal sphincters, as it tones up the parts and relaxes the spasm. There is scarcely any pain, as by slowly introducing the vibratode, the nerves are anesthetized. Stimulation of the third and fourth sacral nerves is very beneficial in some of these cases.

I have employed vibratory stimulation in a few cases of anal pruritus where I was unable to locate the exciting cause, but without benefiting the patient.

In my experience, the greatest field of mechanical vibratory stimulation for the proctologist is in the treatment of chronic constipation, and here the results obtained are certainly remarkable. As constipation is often the result of faulty secretion and excretion of the stomach, liver and spleen, also of the small and large intestines, with a weakened or absent peristalsis, we turn our attention to each of these organs in its treatment. We stimulate the gastric glands through pressure on the vagi at the sides of the neck. Irritation of these nerves also produces contraction of the stomach, including the pyloric sphincter, hence the vagi are the motor nerves of the stomach. There are also spinal nerves reaching the stomach through the solar plexus, these nerves pass through the spinal cord in the anterior roots of the nerves from the sixth to the twelfth dorsal, passing in the splanchnic nerves to the solar plexus, and thence to the stomach. (Kirke, page 367.)

The activity of the liver may be increased by applying over it interrupted vibration with moderate pressure and a medium short stroke, or by abdominal vibration and spinal stimulation. Dr. Snow, in her book on Mechanical Vibration, pages 214 and 215, says: "Professor Colombo, of Turin, demonstrated in respect to biliary secretion, that 'after ten minutes of trepidation (shaking or vibration) and of tapotement or percussion, the quantity of bile increased considerably in the next four hours. The cholesterin and the biliary soda salts were most abundant. After twenty-five minutes of friction and of petrissage the same results were obtained as after ten minutes of trepidation and tapotement; the maximum result was obtained by combining ten minutes of trepidation and tapotement with ten minutes of friction and petrissage."

The vaso-constrictors of the portal system are the third to the eleventh dorsal, the nerves of the hepatic artery are constrictors contained in the splanchnic, and dilators in both splanchnic and vagus. (Kirke.) From the third dorsal to the first lumbar, the motor nerves of the spleen are given off. The peristaltic movement of the intestines is increased by light stimulations of the vagi in the neck, while a heavy stimulation of the vagi would decrease the peristalsis. The colon, rectum and anus are supplied with nerves from the second lumbar to the fourth sacral. By stimulating these nerve centers, the peristalsis is increased, and the internal sphincter rendered patulous.

In conclusion, I would say that it is necessary to correct any errors in diet, to drink large quantities of water between meals, and at bedtime, and especially to refrain from taking any laxative or cathartic medicine, as we wish to depend upon the physiological action of mechanical vibratory stimulation.

The technique is as follows:—First the patient lies upon the back with the knees well drawn up, thus relaxing the abdominal muscles. The breathing should be slow and deep. I employ the rubber brush attachment, making medium hard pressure. Begin at the ilio-cecal junction, and pass the brush slowly along the ascending, transverse and desending colon. Repeat this procedure four or five times. Lightly stimulate the vagi at the sides of the neck. Then have the patient turn over on the face, extending the arms upward and around the end of the table, thus elevating the ribs. We now use the hard rubber ball attachment, medium stroke and pressure, from the fourth to the twelfth dorsal nerve. The impulse reaches the sympathetic through the posterior primary divisions of the spinal nerves and then along the rami-communicantes. The whole intestinal tract with the exception of the rectum will be influenced by this treatment. Stimulation of the rectum is made with the hard rubber ball attachment, quite heavy pressure and medium stroke, over the third, fourth and fifth lumbar, and second, third and fourth sacral nerves.

Treatments should be given daily until bowels move regularly, then every third day for two or three weeks. As we continue to use mechanical vibratory stimulation, and learn how and when to employ it, I feel confident that as proctologists we will consider it one of our most useful means in relieving and curing our patients.—*Detroit Medical Journal.*

---

Dr. David L. Edsall has been appointed assistant professor of medicine in the medical department of the University of Pennsylvania.

### COCAINE IN MINOR SURGERY.

BY J. HUTCHINGS WHITE, M.D., MUSKOGEE, IND. TER.

Since the anaesthetic properties of cocaine were first demonstrated by Kohler in Berlin, the field of its usefulness has rapidly spread. From its use in minor work on the eye it has ingratiated itself upon us to the extent that major operations are now perfoımed without the use of any other anaesthetic. The more one works with this drug the more one appreciates its value. For minor work it is in most cases superior to either chloroform or ether, producing, when judiciously used, no nausea or vomiting, making it particularly valuable for office work. Cocaine, like other things, however, must be used intelligently in order to estimate its full worth. The prime object is to produce local anaesthesia. This is brought about by the action of the drug on the protoplasm of cells (?). The protoplasm of nerve cells being more sensitive, they are first affected.

Then to produce the quickest and best effects, the drug should be applied to the nerve supplying the field for operation, or to the part directly affecting the terminal filaments. In case of fingers or toes it is not difficult to inject the cocaine laterally into the nerve trunk.

The strength of cocaine to be used depends greatly upon the location of the operative field. The pure crystals to the weakest Schleich solution may be used. In the use of the crystals, I have confined myself to the naso pharyngeal territory. Schleich solutions are:

| | Weak. | Medium. | Strong. |
|---|---|---|---|
| Cocaine | 1-6 grain | 1 1-2 grains | 3 grains |
| Morp. sulp | 1-12 grains | 1-2 grain | 1-6 grain |
| Sod. chloride | 3 grains | 3 grains | 3 grains |
| Boracic acid | 5 grains | 5 grains | 5 grains |
| Boiled water | 4 oz. | 4 oz. | 4 oz. |

A good solution and one which plays a double role, anaesthesia and anaunia, is one containing adrenalin. The action of this solution is slow—due to the adrenalin, but the effects last longer and require very little of the drug cocaine. The solution used by Barker, of London, is:

| | |
|---|---|
| Distilled water | oz. i iss |
| B. Eucain | gr. viii |
| Sol. adrenalin chlor., 1-1000 | mx |

The strength solutions of which I formerly used the most was a 2 per cent. or 4 per cent., but experience has taught me that the same result can be secured by the use of weaker solutions.

Always before using any cocaine solution impress upon your patient the fact that he will experience no pain whatsoever. If you say it will hurt some it will surely pain a great deal and you will encounter trouble.

The method of using cocaine is by means of cotton swabs or hypodermic syringe. In case of mucous membranes or raw surfaces the cotton swab works well. In case of tonsilotomy or tonsilectomy, I use both cotton swab and hypodermic. With the former I infiltrate the path which the knife is to follow. When the crystals are used a cotton swab should be moistened and dipped into them then applied to the surface. Occasionally in the use of the crystals one meets with the untoward effect of the drug, so it is well to be on your guard and interrogate your patient and the moment any disagreeable symptoms are experienced the patient should be made to lie flat upon the back. In a few moments these symptoms disappear and you are enabled to continue your work.

It is to the mucous surface that the stronger solutions are used. These surfaces are moist and secrete more or less fluid, rendering these solutions weaker and consequently less effectual here. With the hypodermic infiltration the weak solutions work admirably.

After injecting a part allow a few moments for the diffusion of the drug before beginning work—a longer time when adrenalin is used—say two to three minutes. In case it is an extremity cocanize the part, allowing a few moments for diffusion before applying tourniquet. In cases where the tourniquet is used or much cocaine used when the operation is complete do not remove the tourniquet entirely, but loosen it for a moment then tighten. Do this several times, allowing a very little of the cocaine to enter the system at a time. By this method you get a free circulation of the cocaine in the part and the application of tourniquet confines the solution and gives you a bloodless field for work.

In operating on inflamed areas, carbuncles, furuncles or abscesses cocaine is our mainstay. We have all been told that the insertion of the needle is as painful as incising the part, but I wish to refute this statement. When you prove to your patient that these painful areas can be incised without pain you will be called upon more than once to show your skill. This is the way you can do it. With a hypodermic syringe loaded with the desired strength of cocaine solution, carefully, for without care you will inflict pain, insert the needle just outside of the inflamed area and cocainize a track in the direction of the center of the inflamed field. Go as far as you can with each prick of the needle, carrying it along under the skin and infiltrating as the needle is passed toward the center of the field. Always after the first prick insert the needle in the cocainized zone, manifest by whiteness of skin, and when the center of inflammation is reached turn the needle inward, cocainizing the deeper inflamed tissues. The line of cocainizing may be carried entirely across the area of inflammation, but it is not often necessary.

In this manner I have opened and curetted with a Volkman spoon large boils and carbuncles with absolutely no pain whatsoever. The first carbuncle I ever operated on after this manner was on the neck of a neurotic patient who had suffered for four days. He suffered no pain after the first prick of the needle and did not know I had opened the carbuncle until the curette reached the bottom of the abscess cavity, a depth of an inch and a quarter, and then the pain was slight.

To preserve the cocaine solution it is well to add a small quantity of boracic acid or a little chloretone.

We should strive to do even minor surgery with the least pain and inconvenience to our patients, and the above method I can assure you will spare many a sufferer additional pain.—*Oklahoma Medical News-Journal.*

---

**This Journal and Atlanta Semi-weekly Journal one year for $1.25 cash in advance. No checks. Strictly to new subscribers.**

### "THE CRIMINAL ALLIANCE BETWEEN THE NEWSPAPERS AND PATENT MEDICINES."

The current number of *Collier's Weekly* prints an important editorial under the above title. To strengthen its utterances the article is given a wide border made up of half-tone-reproduced advertisements clipped from the pages of leading daily papers in several of our most enlightened centers of population. In the same issue of the paper is an editorial which refers to the way in which many of our best cities are systematically infected with typhoid fever—a way which should bring the blush of shame to the face of every city official whose name is on the pay-roll of any of the cities named.

Indeed it is high time that the entire educated America people should rouse from a lethargy, the result of a belief which many have that we are easily the most highly civilized nation on earth. There are many respects in which European countries give us by comparison an appearance which strongly resembles an amount of currency less than a half-dollar which has long been the proverbial standard of cheapness; and this without making a comparison between the death rate from typhoid fever in the Japanese army and our own mortality during the Spanish-American war.

The patent medicine habit has taken firm hold on the people—they are constant druggers—as Mr. Hapgood suggests, old ladies who would indignantly refuse to partake of beer or any other known alcoholic beverage, habitually sustain their flagging spirits with peruna cocktails or safe-cure toddies. In the creation of this national drug-habituation the newspapers have played a principal part. By direct inference, also, for a large part of the wherewithal to be fed and clothed the newspapers are directly beholden to the propriteors of the death-dealing patent medicine.

Retail druggists are right in denying responsibility for this drug-craving that compels them to sell the stuff—and physicians, who are occasionally blamed, have little or nothing to do with it. The manufacturers and the newspapers are to blame—and it is this class of offenders that laws must reach if the evil is to be mitigated.

Incidentally it might be as well to do two things: To enforce the law regarding the advertising and sale of abortifacients and to encourage in every way those daily papers and other periodicals who refuse to become *particeps criminis* by carrying such advertising or who join in the holy war against the criminal manufacturer of poisons especially prepared for self-administration.—*Western Medical Review.*

---

### SULPHATE OF COPPER ALONE, AND IN COMBINATION WITH LIME, FOR THE DESTRUCTION OF MOSQUITO LARVÆ, AS A DEODORANT, AND AS A DISINFECTANT.

A. H. Doty (*Medical Record*, January 21, 1905), reports on the results of a series of experimental tests undertaken to determine the question indicated by the title. It was found that a solution containing one pound of sulphate of copper and one pound of unslacked or rock lime (calcium oxide) in ten gallons of water was promptly effectual in causing the death of mosquito larvæ when added in the proportions of one gallon of solution to fifty gallons of the infected water. Solutions of copper or of lime alone were less satisfactory. The result is not due to a toxic action of either of the chemicals, but to the fact that a precipitate is formed which rapidly removes from the water the organic matter upon which the larvæ depend for nourishment and life. This method is applicable only in collections of stagnant and offensive water, where it not only destroys the larvæ, but also deodorizes the fluid; but in swamps or bodies of water covering large areas, other measures are preferable. As a deodorant, the mixture of copper and lime in the proportions stated is the most valuable and practical agent we possess for the purpose. Its action is rapid and permanent; it is practically harmless; is cheap and easily made, and can be employed equally well for deodorizing solids or fluids. The experiments on the germicidal properties of copper sulphate show that it has possibilities as a disinfectant, but no definite statements can as yet be made.

---

An international exhibit of hygiene will by request of the Italian general health department be added to the exhibition at Milan in 1906.

### A FEW REMARKS ON THE TREATMENT OF TUBERCULAR EXACERBATIONS.

DR. R. D. MUSSEY, Glendale, Ohio.

Exacerbations are a common feature of preliminary tuberculosis as every one knows who has had much experience with these cases. Indeed, they are almost characteristic of the disease. A tuberculosis patient may get along quite comfortably for weeks, and even months, without treatment, suffering very little and with little or no loss of weight. Sooner or later, however, over exertion and error in diet or some unknown cause brings on what seems like a bad cold or an attack of grippe or some such symptom. Then the temperature and cough grow worse, loss of strength and flesh go on rapidly and the patient either dies of the attack or makes an imperfect recovery, to go much as before the attack, but upon a lower physical plane. The more advanced the lesion, the more severe and frequent the exacerbations. In the treatment of many cases I have found that they are most successfully handled as follows:

The patient is put to bed upon an exclusive milk and Bovinine diet, the quantity of milk and Bovinine is rapidly increased until the patient is taking from four to five quarts of milk and from four to six ounces of Bovinine each day. Under this complete and full nutrition better results can be obtained than by any other line of treatment.

---

### NUTRITION IN HEART LESIONS.

T. J. BIGGS, M.D., Stamford, Conn.

A most essential factor in the handling of cardiac lesions, either functional or organic, is proper nutrition. In fact, I know of no other disease where failure attends the proper selection of remedies so often as in cardiac trouble, the cause being due to improper feeding. In functional derangements, the most carefully selected remedies fail to bring about the desired result if the diet of the patient be improper or he be overfed, while in structural lines we are defeated before we begin if we neglect the nutrition. When we take into consideration that nearly every functional disturbance of the heart, in perhaps 40 per cent. or more cases, can be traced directly to gastric intestinal disturbance, we will all the more rightly appreciate the great importance of a proper diet. In handling these nervous conditions or functional abnormalities, one can accomplish very little with medicine unless his patient's diet is properly selected. The diet should be one requiring little or no digestion and yet supplying in proper proportions a full quantity of the elements of an absolute nutrition. When we come to prescribe for structural lesions, we find the diet most important, for every organic lesion is not only aggravated by faults of digestion, but the nutrition of the organ itself relies largely upon the diet furnished. If we take into consideration the various valvular derangements, it will be found that the heart is able to do its work owing to compensatory changes that have gradually taken place, and that the case only becomes grave when compensation fails.

The chief aim in the treatment should be to maintain this stage of compensation and while the drugs usually employed in these conditions bring about desired results in part, complete results are not obtained unless proper and complete nutrition is supplied. In cardiac enlargement the same state of affairs prevails. To maintain the heart, when hypertrophied, a uniform degree of nutrition is most essential. In atrophy and dilatation we should endeavor to build up the heart-tissue and such food as will supply the necessary elements should be selected. In fatty conditions of the heart a proper diet is most essential and should receive the physician's careful attention.

In the handling of all cardiac conditions, and my experience has been a large one, I have found that Bovinine was the ideal food and tonic. It does not over-stimulate the heart but supplies sufficient stimulation. It gives to the system a proper proportion of every element of nutrition and a normal amount of assimilable iron. Each individual case must be studied and the quantity of Bovinine administered suited to that case.

---

READERS are requested to see advertisement of the Medical Department University of Louisville, on page XXIII.

